# Operative Management of Avascular Necrosis of the Femoral Head in Skeletally Immature Patients: A Systematic Review

**DOI:** 10.3390/life12020179

**Published:** 2022-01-26

**Authors:** Filippo Migliorini, Gerardo La Padula, Francesco Oliva, Ernesto Torsiello, Frank Hildebrand, Nicola Maffulli

**Affiliations:** 1 Department of Orthopaedic, Trauma and Reconstructive Surgery, RWTH University Hospital, 52074 Aachen, Germany; fhildebrand@ukaachen.de; 2 Department of Medicine, Surgery and Dentistry, University of Salerno, 84081 Baronissi, SA, Italy; gerardo.lp@outlook.it (G.L.P.); olivafrancesco@hotmail.com (F.O.); ernesto.torsiello94@gmail.com (E.T.); n.maffulli@qmul.ac.uk (N.M.); 3 Faculty of Medicine, School of Pharmacy and Bioengineering, Keele University, Stoke on Trent ST4 7QB, UK; 4 Centre for Sports and Exercise Medicine, Barts and the London School of Medicine and Dentistry, Queen Mary University of London, Mile End Hospital, London E1 4DG, UK

**Keywords:** osteonecrosis, femoral head, skeletally immature

## Abstract

Purpose: Osteonecrosis of the femoral head (ONFH) is common in skeletally immature patients. The management of ONFH is controversial, with limited evidence and unpredictable results. This study systematically reviewed the current operative modalities and clinical outcomes of surgical management for ONFH in skeletally immature patients. Methods: The present study was conducted according to the PRISMA 2020 guidelines. PubMed, Google Scholar, Embase, and Web of Science databases were accessed in October 2021. All the published clinical studies reporting data concerning the surgical management of ONFH in skeletally immature patients were included. Results: This review included 122 patients (127 hips). 38.2% (46 of 122) were female. The mean age of the patients was 14.2 ± 2.3 years. The mean duration of the follow-up was 55.3 ± 19.6 months. The Harris Hip Score improved from 68.8 ± 11.9 at baseline to 90.5 ± 6.5 at last follow-up (*p* < 0.0001). Femoral head collapse and secondary hip degeneration were the most common complications. Conclusion: Several surgical techniques are available and effective for the management of ONFH in skeletally immature patients. This study evidenced high heterogeneity of the surgical procedures and eligibility criteria. Further high-quality investigations are required to establish proper indications and surgical modalities.

## 1. Introduction

Osteonecrosis of the femoral head (ONFH) is a disabling condition which leads to progressive pain, deformity, and early-onset osteoarthritis [1,2]. The growth plate acts as a barrier for the intraosseous blood vessels, and femoral head vascularisation is guaranteed by the lateral epiphyseal vessels [3,4,5]. Once bone maturity has been reached, the lateral epiphyseal vessels, the vessels of the metaphysis, epiphysis, and round ligament of the femur anastomose [3,4,5,6]. The aetiology of ONFH can be traumatic and atraumatic [7]. In traumatic ONFH, a fracture may cause an interruption of the blood supply to the femoral head [8]. Although proximal femur fractures in skeletally immature patients are rare, the risk of ONHF insurgence is high, and affects approximately 20% of such patients [9,10,11,12]. Several causes of atraumatic ONFH in the young population have been described; prolonged steroids consumption [7,13,14] and Legg-Calvé-Perthes disease [15,16] represent the most common etiological factors; sickle cell disease [17,18], lymphoblastic leukaemia [19,20], human immunodeficiency virus (HIV) infection [21,22] and Gaucher’s disease [23] are other less common causes. The management of ONFH in skeletally immature patients is controversial, with limited evidence and unpredictable results [24]. Several operative strategies have been developed to regenerate or avoid further femoral head collapse, but no consensus has been reached. This study systematically reviewed the current available evidence on the surgical management of ONFH in skeletally immature patients, focusing on techniques, efficacy, and the safety profile.

## 2. Material and Methods

### 2.1. Eligibility Criteria

All of the clinical studies evaluating the efficacy and safety of operative management of ONFH in skeletally immature patients were considered. According to the authors language capabilities, articles in English, German, and Italian were eligible. Only studies published in peer-reviewed journals were eligible. Only level I to IV of evidence articles, according to Oxford Centre of Evidence-Based Medicine [25], were considered. Reviews, opinions, letters, and editorials were not considered. Animals, biomechanics, computational, and cadaveric studies were not eligible. Only studies investigating a population with a mean age ≤18 years were eligible. Studies which involved less than five patients were excluded, as were those with a mean follow-up ≤24 months, or studies focusing on Legg-Calve-Perthes disease. Only studies which reported quantitative data under the outcomes of interest were considered.

### 2.2. Search Strategy

This systematic review was conducted according to the Preferred Reporting Items for Systematic Reviews and Meta-Analyses: the 2020 PRISMA statement [26]. The following algorithm was preliminary pointed out:

- Problem: ONFH;
- Intervention: operative management;
- Comparison: operative techniques;
- Outcomes: efficacy and safety profile;
- Timing: minimum 24 months follow-up;
- Age: ≤18 years.

In October 2021, the following databases were accessed: Pubmed, Web of Science, Google Scholar, EMBASE. No time constrains were used for the search. The following keywords were used: osteonecrosis, avascular, necrosis, collapsed, femur, femoral, head, fractures, ONFH, adolescent, paediatric, skeletally immature, surgery, operative, treatment, management, osteotomy. The keywords were combined using the Boolean operator AND/OR.

### 2.3. Selection and Data Collection

Two authors (F.M.; G.L.P.) independently performed the database search. All resulting titles were screened and the abstract of the articles which matched the topic were accessed. If the abstract matched the topic, the full-text article was accessed. A cross-reference of the bibliography of the full-text articles was also undertaken. Disagreements were debated and the final decision was made by a third author (N.M.).

### 2.4. Data Items

Two authors (F.M.; G.L.P.) independently performed data extraction. Generalities of the articles were retrieved: author and year, journal, duration of the follow-up, type of study. The following data were extracted: age, sex, aetiology, type of treatment, Harris Hip Score (HHS) [27], and complications.

### 2.5. Study Risk of Bias Assessment

The Coleman Methodology Score (CMS) was used to assess the quality of the methodological assessment [28]. Each article was assessed by a single reviewer (G.L.P.). The CMS is divided in two sections: A and B. Section A evaluated seven items: study size (number of patients), mean follow-up (from <12 month to >60 month), number of surgical approaches, type of study (randomized, prospective, or retrospective), and the description of diagnosis, surgical procedure, and rehabilitation. Section B evaluated the outcome criteria, method of assessing outcomes, and subject selection process. The final score ranges from 0 to 100 points, with values > 60.

### 2.6. Synthesis Methods

All statistical analyses were performed using the IBM SPSS Software version 25. For descriptive statistics, mean and standard deviation were calculated. To evaluate the improvement of the HHS from baseline to the last follow-up, the mean difference (MD) and T-test were performed. The confidence interval (CI) was set at 95%. Values of *p* < 0.05 were considered statistically significant.

## 3. Results

### 3.1. Study Selection

**Figure 1:**
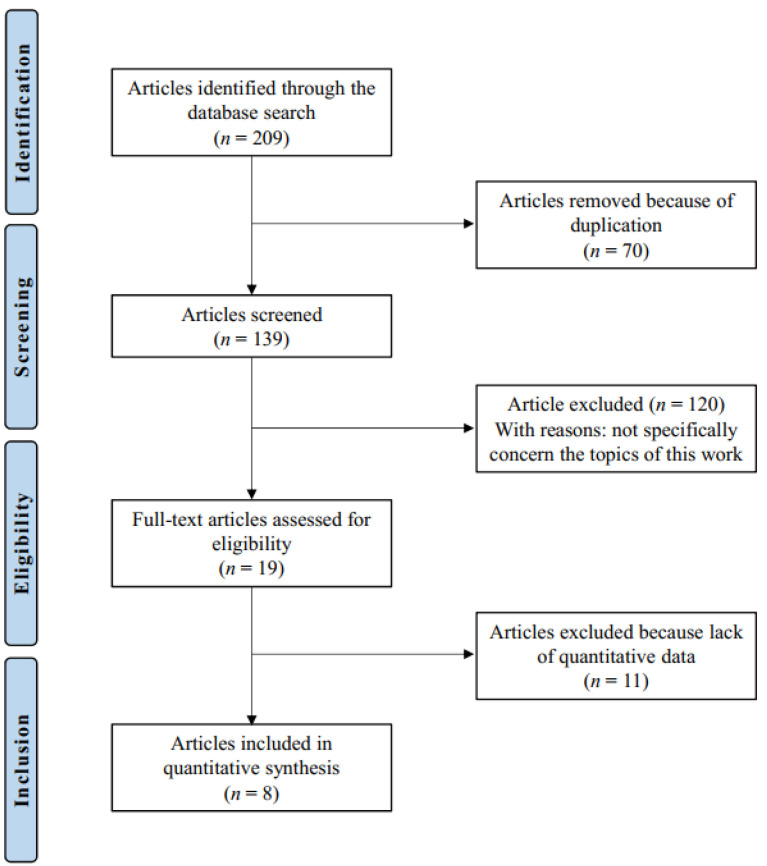
Flow chart of the literature search.

The initial literature search resulted in 209 studies. Of them, 70 were excluded for being duplicates. Another 120 were not eligible, either for not matching the topic (*N* = 71), focusing on adults (*N* = 22), focusing on surgical management (*N* = 6), focusing on Legg-Calve-Perthes disease (*N* = 16), the type of study (*N* = 2), full text not accessible (*N* = 2), or uncertain results (*N* = 1). This left 19 articles for inclusion. An additional 11 studies were excluded as they did not report quantitative data under the outcomes of interest. This resulted in eight studies for analysis. The literature search results are shown in Figure 1.

### 3.2. Study Risk of Bias Assessment

The length of the follow-up was acceptable in most studies. Surgical technique, diagnosis, and rehabilitation protocols were generally well described. The study size and the retrospective design of most of the included studies represented the main limitations highlighted by the CMS. Outcome measures, assessment timing, and selection process were also clearly defined by most studies. Finally, the mean methodology score of 73.8 (range: 69 to 79) suggested an overall good quality of the methodological assessment (Table 1).

### 3.3. Patient Demographics

Data from a total of 120 patients (125 hips) were collected; 38.3% (46 of 120) were females. The mean age of the patients was 14.2 ± 2.3 years. The mean follow-up was 55.3 ± 19.6 months. The aetiology of ONFH was: 72.8% (91 of 125) traumatic, 17.6% (22 of 125) caused by sickle cell disease, 3.2% (4 of 125) idiopathic, and 4.8% (6 of 125) other (infection, steroids use, lymphocytic leukemia, post-adrenal tumor excision). The demographic data are shown in Table 2.

### 3.4. Efficacy

Five articles [2,29,30,32,35] reported data concerning the Harris Hip Score data. Irrespective of the surgical technique, the mean HHS improved from 68.8 ± 11.9 at baseline to 90.5 ± 6.5 at last follow-up (+21.7; 95% CI 20.06 to 23.33; *p* < 0.0001). Pre- and post-operative plan radiographies [2,29,30,31,32,33,34,35] and magnetic resonance imaging (MRI) [2,29,30] were evaluated. All surgical techniques were effective in regenerating the femoral head or in blocking the progression of degeneration [2,29,30,31,32,33,34,35]. Improvement in range of motion and pain relief was also observed [2,29,30,31,32,33,34,35]. THA and femoral osteotomies have been used to correct leg shortening [2,29,30,33]. The efficacy of each surgical technique is reported in greater detail in Table 3.

### 3.5. Complications

Failure of treatment to prevent the progression of femoral head collapse, secondary hip degeneration, and reoperation were the main complications [2,29,30,31,32,33,34,35]. These complications were reported in 46 of the 120 patients analysed (38.3%). 54.3% (25 of 46) of the patients who experienced complications were treated conservatively. Failure of treatment to prevent the progression of femoral head collapse was observed in 11 of 100 (11%) surgically treated patients [31,32,33,34,35]. They included the following. One-quarter (5 of 20) of the patients were treated with the trapdoor procedure [31,32], 21.4% (3 of 14) of patients were treated with multiple epiphyseal drilling and autologous bone marrow implantation [34], two of 28 (7.1%) were treated with free vascularised fibular graft [35], and 14% (one of six) of patients was treated with an intertrochanteric osteotomy [33]. Secondary hip degeneration was observed in 18 patients. Of them, 33.3% (6 of 18) were treated with non-vascularized bone grafting via the trapdoor procedure [32]. A total of four patients underwent reoperation. Of them, 18.1% two of 11) had undergone a valgus intertrochanteric osteotomy [2,29], and 14.2% (two of 14) had received multiple epiphyseal drilling and autologous bone marrow implantation [34]. The major complications of each surgical technique are reported in greater detail in Table 4.

## 4. Discussion

According to the main findings of the present study, surgical management is effective to improve symptoms, and decelerate or arrest the evolution of ONFH. Overall pain reduction, motion and gait improvement were reported by most patients. Surgical management aims to revitalize the femoral head, favour spherical remodeling, induce necrosis resorption, and improve hip motion.

Conservative management consists mostly in physiotherapy, bisphosphonates and/or non-steroidal anti-inflammatory drug (NSAID) administration, vitamins supplementation, weight-bearing limitation, and, if possible, removal of the underlying cause [17,36,37,38,39]. These therapeutic measures demonstrated limited potential in stimulating femoral head remodeling, resorption of necrotic tissue, or improvement of hip motion. Most conservatively managed patients evidenced worsening of the HHS, progression of the osteonecrosis, and further joint degeneration [32,40,41]. Irrespective to the surgical strategy, the mean HHS significantly improved by approximately 21%. This improvement overcomes the minimally clinical important difference (MCID) [2,29,30,32,35,42,43].

The mean age of the population included for analysis was 14 years. At the approximate age of 14 years in girls and 16 years in boys, the growth plates of the femur start to fuse [44,45]. Hence, the population included was likely to still have open epiphyseal growth plates. Patients with open epiphyseal growth plates demonstrated higher bone marrow derived stem cell concentration compared to adults [46,47,48]. Moreover, the open physis promotes greater self-healing [49]. In this context, the use of bone marrow stimulating techniques should promote a greater regenerative potential in this population compared to adults. Further studies investigating the efficacy of bone marrow stimulating techniques of simple execution (e.g., isolated core decompression) in early ONFH should be considered. Multiple epiphyseal drilling augmented with autologous bone marrow implantation has been investigated in skeletally immature patients with ONFH secondary to sickle cell anaemia [34]. 29% (four of 14) of patients treated with multiple epiphyseal drilling augmented autologous bone marrow demonstrated significant improvements in symptoms and motion, and no further necrosis progression was evidenced in the half of the patients (seven of 14) [34]. Valgus and varus intertrochanteric osteotomy and triple acetabular osteotomy have been successfully performed in the early stages of ONFH to promote bone healing by discharging weight bearing over the necrotic area [2,29,30]. In patients with moderate to advanced ONFH, bone grafting may be performed [50,51,52]. Bone grafting replaces necrotic material, stimulates revascularisation, and, given its osteoinductive properties, promotes bone reconstruction [35,52,53]. The trapdoor procedure was effective in reducing the risk of femoral head collapse progression, improving clinical function in patients with moderate ONFH [32,54]. The trapdoor procedure aims to replace the necrotic area, promote revascularisation, and prevent collapse [31,54]. Ko et al. [31] investigated the potential of the trapdoor procedure in isolation and in combination with femoral and/or acetabular osteotomy on 13 young patients with advanced ONFH. The isolated procedure failed in two of three patients, while those operated with the combined procedure reported significant improvement in symptoms, activity, and range of motion [31]. These results indicated that the isolated procedure may be recommended for early-stage degeneration, and should be performed in combination in more advanced stages of ONFH. Zhang et al. [35] reported the outcome of 28 skeletally immature patients treated with a free vascularised fibular graft. Regardless of the extent of ONFH, all patients experienced an excellent outcome, with an improvement in HHS from 60.4 to 94.2 points on average [35]. In patients with advanced ONFH, THA improved joint function and symptoms, restored leg length difference, and led to the greatest improvement of the HHS [35]. However, despite its excellent results, THA should be performed only as last resort, as long-term survival of the implants is not ensured [55,56,57].

Current evidence on ONFH management refers almost exclusively to the adult population or mixed cohort populations. Rotational osteotomies, core decompression in isolation or in combination with vascularised bone grafts, bone morphogenetic proteins, bone marrow-derived cells, mesenchymal stem cells, or combinations of the above, are the most commonly reported surgical procedures performed for early to moderate ONFH [58,59,60,61,62,63,64,65,66]. Total hip replacement is recommended for end stage ONFH or in cases of failure of the index procedures [67,68,69]. Core decompression augmented with autologous bone marrow-derived transplantation or combined with autologous bone graft are effective in reducing symptoms of ONFH and the need for arthroplasty in the adult population [63,64]. The regenerative potential of autologous mesenchymal stem cells have been poorly investigated in the younger population, and further investigations are required.

This study certainly has limitations. The reduced number of included studies and consequently analysed procedures do not allow for the provision of accurate evidence-based recommendations. The cohort of included patients is not homogeneous in terms of pathogenesis of ONFH. Proximal femur fractures [2,29,31,32,33,35], sickle cell anemia [30,31,34], idiopathic causes [31], infection [33], steroid use [31], lymphocytic leukemia [31], and post-adrenal tumor excision [31] are the most common causes of ONFH reported. With regard to proximal femur fractures, transepiphyseal, transcervical, cervicotrochanteric, and intertrochanteric fractures were reported. Furthermore, most authors used the HHS for patient evaluation. However, this score has not been validated for skeletally immature patients. When considering paediatric populations, child-friendly patient reported outcomes measures (PROMs) should be used. Most of the included studies were retrospective, with the lack of a control group and limited information. Given the lack of quantitative data, bilaterality of ONFH it was not possible to investigate separately. Finally, articles concerning Perthes disease have been excluded, as the management of these patients has been extensively analysed in the literature [70,71,72,73,74,75,76,77,78,79,80,81,82], and evidence on non-Perthes related ONFH is limited. Two studies which have been included for analysis reported data on patients with Perthes disease [31,33]; however, since the authors reported the results separately, data from patients with Perthes disease were not considered. Ko et al. [31] included for analysis 12 patients (13 hips). Of them, nine patients (nine hips) were affected by Perthes disease, and three patients (four hips) by idiopathic ONFH. In the present study, only the patients with idiopathic ONFH were considered for analysis. Current evidence on the surgical management of ONFH in skeletally immature patients is hence very limited. Therefore, larger studies should be undertaken to establish clearer indications and protocols in the paediatric population.

## 5. Conclusions

Several surgical techniques are available and effective for the management of ONFH in skeletally immature patients. This study evidenced high heterogeneity of the surgical procedures and eligibility criteria. Further high-quality investigations are required to establish proper indications and surgical modalities.

## Figures and Tables

**Table 1:** Methodological quality assessment.

Endpoint	Mean	Range
Part A: only one score to be given for each of the 7 sections		
Study size: number of patients	1	0 to 4
Mean follow-up	7.75	4 to 10
Surgical approach	8.87	7 to 10
Type of study	3.75	0 to 10
Descriptions of diagnosis	5	5 to 5
Descriptions of surgical technique	10	10 to 10
Description of postoperative rehabilitation	5	5 to 5
Part B: may be given for each option in each of the 3 sections		
Outcome criteria	10	10 to 10
Procedure of assessing outcomes	13	12 to 15
Description of subject selection process	9.37	5 to 10

**Table 2:** Generalities and patient demographic of included studies.

Author, Year	Journal	Study Design	Purpose	Participants (*n*)	Mean Age	Female (%)	Follow-Up (Mean Months)
Bartoníček et al., 2011 [2]	*Arch. Orthop. Trauma Surg.*	Prospective	Medium-term outcome assessment of valgus-flexion intertrochanteric osteotomy for ONFH after slipped capital femoral epiphysis	5	12.5	40	73
Bartoníček et al., 2012 [29]	*Int. Orthop.*	Prospective	Outcome assessment of post traumatic ONFH treated with total hip replacement or valgus intertrochanteric osteotomy	11	17	82	89
Gatin et al., 2016 [30]	*Haemoglobin*	Prospective	Medium-term outcome assessment of triple acetabular osteotomy or femoral varus osteotomy for ONFH in sickle cell patients	10 (11 hips)	10.2	50	63.6
Ko et al., 1995 [31]	*J. Paed. Orthop.*	Retrospective	Report outcome of trapdoor bone grafting procedure combined with containment osteotomy of femur and acetabulum	12 (13 hips)	15.5	46	53
Li et al., 2018 [32]	*Int. J. Clin. Exp. Med.*	Retrospective	Compare the outcome of non-vascularized bone grafting via trapdoor procedure in traumatic collapsed-stage ONFH with conservative methods	37	15.1	27	44.5
Nötzli et al., 1995 [33]	*J. Paed. Orthop.*	Retrospective	Report outcome of extruded femoral head reorientation with combined open reduction and intertrochanteric osteotomy in Ficat stage III and IV ONFH	6	14.5	14	46
Novais et al., 2015 [34]	*J. Paed. Orthop.*	Retrospective	Results of Multiple Epiphyseal Drilling and autologous bone marrow implantation for ONFH secondary to sickle cell disease in children	11 (14 hips)	12.7	18	25
Zhang et al., 2011 [35]	*J. Bone Joint Surgs.*	Retrospective	Free vascularised fibular graft for post-traumatic osteonecrosis of the femoral head in teenage patients	28	16.3	28	48

**Table 3:** Efficacy of each surgical technique.

Author, Year	Intervention	Efficacy
Bartoníček et al., 2011 [2]	Valgus-flexion intertrochanteric osteotomy (five hips)	In all patients, the osteotomy healed within three months without complications. Limb shortening was fully corrected in all the patients. Radiographs and MRI scans after osteotomy proved resorption of the necrotic segment of the femoral head and its remodeling in all the patients.
Bartoníček et al., 2012 [29]	Valgus intertrochanteric osteotomy (six hips); Total hip replacement (five hips)	VITO: in five of six patients osteotomy healed within three months without complications. Leg shortening was fully corrected in four of six patients. MRI scans after surgery showed resorption of the necrotic segment of the femoral head and its remodeling in all six patients. THR: all patients healed without complications. Shortening of the affected limb was compensated in all patients. The range of movement was only minimally limited compared with the contralateral side. All patients were highly satisfied. No signs of implant loosening were noted at the final follow-up.
Gatin et al., 2016 [30]	Triple acetabular osteotomy (seven hips); Varus osteotomy (2 hips); Combination of both (two hips)	All patients had an objective functional improvement in terms of pain and range of motion. X-ray and MRI showed bone reconstruction in all the patients. At the last follow-up, joint congruency was satisfactory for all patients. No postoperative complications related to the surgery were reported.
Ko et al., 1995 [31]	Non-vascularised bone grafting via trapdoor procedure combined with containment osteotomy of femur and acetabulum (14 hips)	On clinical evaluation all patients had improvement in pain, activity, and hip motion. The pain score improved from 2.3 to 4.6. The activity score improved from 1.8 to 4.5, and the motion score from 2.8 to 3.9
Li et al., 2018 [32]	- Non-vascularized bone grafting via trapdoor procedure	Surgery provided a lower risk of femoral head collapse progression, improved clinical function, and prevented rapid hip degeneration compared to conservative treatment
Nötzli et al., 1995 [33]	- Open reduction and intertrochanteric osteotomy	Postoperative joint motion improves in four of the six patients; all six patients had widening of the joint space and improved congruency at last follow-up radiographs; all six patients had improvement in gait
Novais et al., 2015 [34]	- Multiple Epiphyseal Drilling and Autologous Bone Marrow Implantation	Four of 14 hips had radiologically improvement. No further progression of the necrotic process was observed in seven of 14 hips at the latest follow-up. Pain relief and improvement in range of motion was also observed in all the patients
Zhang et al., 2011 [35]	- Free vascularised fibular graft	No patient had complication at the site of the fibular graft harvesting. No patients required conversion to a total hip replacement. 22 of 28 hips (79%) improved radiologically, whit sign of resorption of the necrotic segment of the femoral head and its remodeling. Pain relief and improvement in range of motion was observed in all the patients

**Table 4:** Main complications of each surgical technique.

Author, Year	Intervention	Complications
Bartoníček et al., 2011 [2]	Valgus-flexion intertrochanteric osteotomy (five hips)	One of the five patients was performed another osteotomy at one year after the surgery for the presence of a necrotic segment of the femoral head
Bartoníček et al., 2012 [29]	Valgus intertrochanteric osteotomy (six hips); Total hip replacement (five hips)	VITO: one of six patients was reoperated five months after osteotomy for nonunion THR: no complications were observed
Gatin et al., 2016 [30]	Triple acetabular osteotomy (seven hips); Varus osteotomy (two hips); Combination of both (two hips)	In one patient, femoral osteotomy was performed in addition to a triple acetabular osteotomy. Pneumonia occurred in a patient at two days postoperatively
Ko et al., 1995 [31]	Non-vascularized bone grafting via trapdoor procedure combined with containment osteotomy of femur and acetabulum (13 hips)	Trapdoor procedure failed in two patients, one patient was subsequently treated by THA and the other one by femoral head allograft, at three and two years, respectively. Several patients had a mild to moderate limp but did not have pain. One patient at the latest follow-up had a 3.2 cm limb length discrepancy
Li et al., 2018 [32]	Non-vascularized bone grafting via trapdoor procedure	In the surgical group the progression of femoral head collapse was found in three of 17 patients, secondary hip degeneration was found in six of 17 patients. In the conservative group, progression of femoral head collapse was found in 13 of 20 patients, secondary hip degeneration was in 12 of 20 patients
Nötzli et al., 1995 [33]	Open reduction and intertrochanteric osteotomy	Treatment failed in one patient, his hip subluxed, causing loss of motion and ongoing destruction of the femoral head. The patient also developed pain and his limp was more pronounced
Novais et al., 2015 [34]	Multiple Epiphyseal Drilling and Autologous Bone Marrow Implantation	Three hips had disease progression and two patients have required subsequent surgical procedures at latest follow-up
Zhang et al., 2011 [35]	Free vascularised fibular graft	Radiologically, four of 28 hips were not improved and two of 28 hips were worse

## Data Availability

The datasets generated during and/or analysed during the current study are available throughout the manuscript.

## Abbreviations

Harris Hip Score (HHS), Coleman Methodology Score (CMS), osteonecrosis of the femoral head (ONFH).
